# Lessons Learned in Transgender Peer Navigation: A Year of Reflective Journaling

**DOI:** 10.3390/ijerph22050678

**Published:** 2025-04-25

**Authors:** Gwen Rose, Ken Mullock, Elijah Gatin, T. Fayant-McLeod, Michelle C. E. McCarron, Megan Clark, Stéphanie J. Madill

**Affiliations:** 1 School of Rehabilitation Science, University of Saskatchewan, 104 Clinic Pl, Saskatoon, SK S7N 2Z4, Canada; gwen.rose@usask.ca; 2 Trans Sask Support Services, 429 9th St E, Prince Albert, SK S6V 0Y1, Canada; kjm824@usask.ca (K.M.); navigator.north@transsask.ca (E.G.);; 3 Centre for Kinesiology, Health and Sport, University of Regina, 3737 Wascana Parkway, Regina, SK S4S 0A2, Canada; michelle.mccarron@uregina.ca; 4 Academic Family Medicine, College of Medicine, University of Saskatchewan, 3311 Fairlight Dr, Saskatoon, SK S7M 3Y5, Canada

**Keywords:** transgender, health services, equity-deserving populations, peer navigators, reflective journaling, program evaluation

## Abstract

People who are trans and gender-diverse are underserved by the healthcare system; one way to improve healthcare access is with peer healthcare navigators. We piloted two trans peer health navigators from April 2021 to March 2022 in a small Canadian province. The purpose of this study was to explore how trans peer navigators experienced their work and work environment through reflective journalling. The navigators journalled roughly weekly. They were encouraged to interrogate their own biases and to think about what was omitted from conversations with others. Each journal was treated as a qualitative case study, anonymized and analyzed thematically using Interpretive Phenomenological Analysis. Six themes emerged: expected work, unexpected work, teamwork, lived experience, challenges, and systemic factors. These themes were complexly interwoven with a network of subthemes that frequently fell under multiple main themes and were highly emotionally charged, many both positively and negatively. The importance of navigators being transgender themselves was highlighted. The rewards came from being able to provide meaningful help to people in their community and the challenges came from not being respected by other healthcare providers and systemic barriers that prevented them from helping clients. The navigators successfully adapted their services to bridge some systemic barriers. This research has implications for improving both navigators’ and clients’ experiences.

## 1. Introduction

### 1.1. Theoretical Background

People who are transgender and gender-diverse (TGD) are a medically underserved community, who frequently report barriers to accessing healthcare [1]. Scheim et al. found that among 2217 trans and non-binary respondents to their survey, 81.4% had a primary healthcare provider (HCP); however, only 52.3% were comfortable discussing trans health issues with their HCP and 44.4% had an unmet healthcare need [1]. In Saskatchewan, a survey of 188 primary HCPs found that while 96% felt comfortable providing general healthcare to people who are TGD, only 30% felt comfortable looking after individuals’ transition-related medical needs [2]. Navigation has been successful in helping numerous equity-seeking groups, including people who are TGD, children and youth with mental health problems and addictions, and Indigenous people with cancer, improve their access to, and quality of, healthcare across Canada [3]. Our project, the Transgender Research And Navigation Saskatchewan (TRANS) Project, was born out of a desire to address the healthcare needs of the TGD community in Saskatchewan: the project was intentionally developed as a collaboration between healthcare and research professionals and members of the TGD community, foregrounding the lived experience of the latter (and including some people who are TGD who are also researchers). The TRANS navigator service was modelled on the services provided by Trans Care BC [4]. We were interested in seeing if peer navigators for the TGD community in Saskatchewan could do for this community what research has shown that navigators have done for numerous other underserved groups of people.

### 1.2. Our Project’s Work

We piloted two navigators—both of whom are TGD—from April 2021 to March 2022. In addition to being members of the TGD community, it was a job requirement that the navigators also have healthcare or TGD non-profit experience. The navigators were employed by the study and were housed in 2SLGBTQ+ community-based organisations (CBOs) in Regina and Saskatoon, where they had a mix of in-person and remote work. One navigator provided services for the city of Regina and southern Saskatchewan. The other navigator provided services for the city of Saskatoon and northern Saskatchewan. These services were varied: for clients who are TGD they provided information and resources, worked to connect clients with appropriate healthcare providers (HCPs), and helped clients complete and submit forms to change their legal name and/or gender-marker (becoming notaries public to provide this service), among other services. When working with HCPs, the navigators provided information and/or resources about trans health needs and care, connected HCPs interested in trans health to more experienced providers or advised them on client care (e.g., how to refer for gender-affirming surgeries), advocated on behalf of clients, and more. The navigators also conducted educational sessions for groups. Most services were provided by email, telephone, or videoconferencing due to COVID-19 health restrictions. Clients and healthcare providers (HCPs) self-referred to the navigators, and clients and HCPs in turn served as word-of-mouth advertising for the navigators’ services, in addition to posters in HCPs’ offices or clinics and online means (the navigators’ Instagram^TM^ account, the study’s website, and the websites and social media of partner community organizations). The navigators worked with 299 clients over the year, and 175 provided demographic information. Of these, their mean age was 23.4 ± 8.9 years and 40% were boys, men, or trans men, 40% were girls, women, or trans women, and 20% were non-binary.

### 1.3. Reflective Journalling and Research Questions

We asked the navigators to reflect regularly in journals throughout the pilot period to avoid the recall difficulties [5]. While our team’s decision to employ reflective journaling predated the following literature, we note that a concurrent study also used reflective journaling to analyse the experiences of patient navigators [6]. Murillo-Llorente et al., in a separate study, found that reflective practice journaling (RPJ) “helped [healthcare] students to learn [and] facilitated the acquisition of professional skills and development of values, and improved the self-criticism capacity of students” [7] (p. 10). As Murillo-Llorente et al. further noted, RPJ is most often used in educational settings [7], but we believed that it would be appropriate for use as the navigators developed their roles over the course of the pilot year, allowing them to evaluate their own experiences and practice in real time, whereby they could immediately implement changes and improvements in their work that were inspired by their reflective practice.

As a research team, we analyzed their journals to answer the following questions: (1) how did the navigators themselves experience their work, the environment(s) in which they worked (in particular the healthcare system and CBOs), and their roles and biases? and (2) what emerged during the year that the navigator project was piloted that we had not anticipated? This could include work tasks, barriers or facilitators to the work, relationships, or anything else salient to the navigators. This paper explores our analysis of the navigators’ reflective journals.

We further evaluated the navigator pilot project by conducting baseline focus groups of Saskatchewan residents who are TGD on healthcare access [8], post-service interviews with HCPs and clients who worked with the navigators [9], and post-service client surveys [10], which have been or will be reported on elsewhere.

## 2. Materials and Methods

### 2.1. Participants

To understand the two navigators’ working experiences, we asked them to keep journals over the course of the pilot period (April 2021–March 2022). Only the navigators employed in the pilot were included in the study as we were interested in evaluating the program from multiple angles, including usage statistics, post-service use surveys, client and HCP interviews, and the navigators’ perspectives [8,9,10]. The inclusion criteria were the same as the employment criteria: the individual had to be TGD, and they had to have either healthcare or CBO experience. The navigators were both researchers and participants, and they provided written informed consent when they were hired.

The navigators could keep their journals in the form (physical or digital) they preferred; both chose digital formats. All names were anonymized within the documents. The navigators are also not named here; their journals are attributed only to “Navigator A” or “Navigator B”: the intent of this is to minimize the possibility of identifying any individuals referred to, however obliquely, in quotations from the journals, by making it unclear exactly which navigator, in which region, is quoted.

### 2.2. Materials

The navigators were encouraged to reflectively interrogate their own biases in their interactions with both clients and HCPs. They were asked to think about what might be missing from these conversations: what the people who are TGD and HCPs do and do not ask or say. Researchers stressed to the navigators that there was no wrong or right way to journal and that the tone, style, and, in particular, narrative of their journal might change from entry to entry or over the course of the pilot period. While some possible prompts were included, the navigators wrote freely. Navigators were asked to journal weekly about recent events to keep their memories fresh.

The journals were not edited by researchers, though quoted excerpts may be edited for clarity, brevity, and spelling or grammatical errors. The journal documents are stored on a secure University of Saskatchewan server accessible only to research team members. Researchers were people who are TGD, academic researchers, or both. The navigators were part of the research team.

### 2.3. Analysis

Given that there were only two data sets (the two journals) and taking into consideration the similarity of the two journals, we treated the journals as a single case study. Navigator A wrote 39 journal entries during the pilot period, while Navigator B wrote 57. This disparity was not meaningful, considering the varying length of the journal entries themselves, and overall, after cross-comparison, all research team members agreed that the journals could be analysed together; the main themes we found were present in both documents.

In analysing the data thematically, we followed the principles of Interpretative Phenomenological Analysis (IPA), given IPA’s “focu[s] on how individuals make sense of their experience of the thing of interest” [11] (p. 1). Engward and Goldspink outline six steps to IPA: (1) read and re-read the data, (2) make initial notes, (3) develop emerging themes, (4) search for connections across these themes, (5) move to the next case, and (6) look for patterns across cases [12] (p. 8). An IPA approach to analysing the journals was important, since IPA encourages researchers to acknowledge their own biases and perceptions of research data [13]. We all knew the navigators well and had heard details of their work before—there was no way to be objective about our analysis of their journals, and, instead, the research team openly discussed their own perceptions of the navigators and their work with the navigators when we met to discuss codes and themes. We encouraged the open discussion of disagreements among researchers and challenged each other to explain why we felt and thought the way we did to increase the awareness of our biases. We followed Engward and Goldspink’s steps as researchers (K.M., E.G., T.F.M., M.M., S.J.M., and M.C.) and completed our analyses of the journals using NVivo 12 software (https://www.qsrinternational.com/nvivo-qualitative-data-analysis-software/home, accessed on 19 January 2024). The initial coding was performed by the navigators who completed the first three steps on their own journals, as did two other researchers per journal. The two smaller teams met to discuss each journal, with an additional researcher (G.R.) who attended both meetings and read both journals but did no initial coding. These steps resulted in the creation of emerging themes; given the navigators’ close personal connections to the data, their personal interpretations of their respective journals were given precedence in this process. In step four, researchers used extended discussions to establish a consensus—if one researcher had coded a theme as “difficult tasks”, for example, and another had coded a theme as “challenging experiences”, these were merged after checking to see if the parts of the journals on which they were based corresponded. Passages could also be double-coded, if researchers had coded them differently and the group agreed that the codes were distinct; for example, “difficult tasks” and “negative feelings” ended up as separate codes. This process was an approach for triangulating the data.

Across both journals, our research team initially created 185 unique codes after merging duplicates. We defined “codes” as terms or phrases that were used to define a meaningful part of the data, as defined by highlighted sentences or paragraphs that contributed to that code. For example, a code titled “end of service” was defined by passages—from both journals—that reflected the navigators’ thoughts on the impending end of the pilot period, after which they would no longer be able to help their clients—this, and not their personal unemployment, was the chief concern of both navigators in reflections relating to this event.

In step five, the two groups exchanged and compared data, including journals and coding, and then all researchers again worked to establish a consensus around the coding for the two journals together, further triangulating codes. In step six, researchers searched for patterns between the two journals, identifying connections across and between the codes. To achieve a more detailed understanding of these connections and patterns, codes that emerged from group discussions were further organized by S.J.M. and G.R. into six larger themes, completing the triangulation, first on a table, then, at the request of the research team, as a graphic to visualize the connections. Quotes selected for this paper for their concise descriptions, illustration, and emotional power were vetted by the navigators, as members of our community whose identities we know we cannot truly anonymize; we wanted to respect their personal lives and their comfort with sharing their recorded thoughts in a public platform. The navigators’ personal connections to this work, and that of research team members, also factored into our analysis of their journals and our commitment to the principle of “nothing about us, without us”. Given our long-established relationships as a team, our practices of open and honest communication, and the inclusion of people with a diversity of positionalities on each small analysis team, we felt assured that we would be able to address each other’s biases as they arose. Simultaneously, these factors allowed our research team to feel confident in our interpretation of the data and with how we portrayed the data in the themes and subthemes.

### 2.4. Ethical Approval

This study was approved by the University of Saskatchewan Behavioural Research Ethics Board (Beh-1897). Maintaining the reflective journals was part of the navigators’ employment contract. All candidates for the navigator positions were informed about the journals during the job interview and the successful candidates provided written informed consent as part of the hiring process.

## 3. Results

### 3.1. Themes

**Figure 1:**
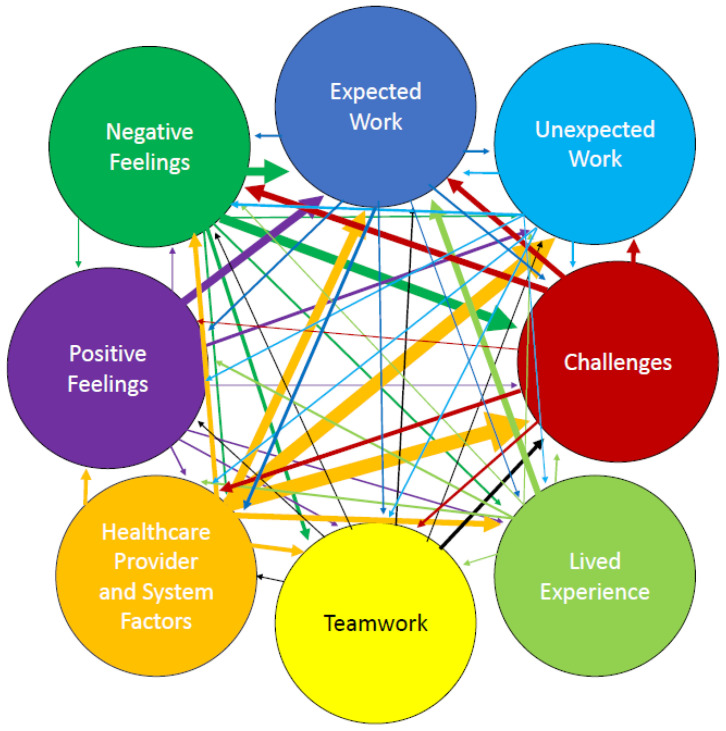
Connections between journal themes. The six themes, plus the emotions evoked by the navigators’ work, are represented by differently colored circles. The interconnections between the themes and emotions are represented by arrows. The thickness of the arrows indicates the frequency with which the two themes were interconnected. An interconnection was recorded when both themes, or the theme and emotion, occurred in the same journal statement.

The 185 codes were the “emergent” themes of the early stage of the analysis; the research team identified six larger themes, expected work, unexpected work, teamwork, lived experience, challenges, and systemic factors, that tied many of the codes together and tasked S.J.M. and G.R. with assessing which of the 185 codes were the most meaningful and organizing them into “subthemes” underneath the six main themes. These themes, and their respective subthemes (some of which overlap), are depicted in full in Table 1. We also cross-coded Positive or Negative Emotions throughout—these “adjacent” or “intertwining” themes reflected the emotional tenor of many of the subthemes and demonstrated the heavily interconnected nature of the themes that emerged from the journals. Figure 1 provides a visual example of how the themes inter-related and how frequently subthemes of one theme were linked to other subthemes of other themes.

#### 3.1.1. Expected Work

This theme was the simplest: its subthemes reference the navigators’ duties that were predicted in the planning stages of the study and are listed on Table 1. The sheer volume of subthemes demonstrates the wide variety of tasks that were expected of the peer navigators. Notably, many aspects of this work—expected or not—were connected to challenges, HCP or systemic factors, or both. For example, many of the journal excerpts making up the subtheme “helping clients to access hormone therapy” were perceived as challenging, and sometimes this was because of HCPs: for example, Navigator A wrote, “I have heard of two doctors in [municipality] and [other municipality] that refused to take on two of my clients because of personal reasons. My clients do not want to cause a fuss, but it is so frustrating to hear those doctors are letting their transphobia get in the way of providing care for people, even if it is not for [hormone therapy]”. Here, the aspect of the work that was perceived as “challenging” also evoked negative emotions in Navigator A, because the navigator was unable to help clients, and, indeed, perceived the clients as having a negative experience as well, through being directly rejected by physicians because of their identity, and still lacked a family physician at the end of the interactions.

#### 3.1.2. Unexpected Work

A surprising variety of tasks that were not foreseen by the research team during planning—that were “unexpected”—arose for the navigators during the pilot period. One subtheme concerned the pure volume of requests from clients, which, as Navigator A noted, contributed to potential burnout: “[t]his week has been overwhelming and very long. I came back from holidays with a large amount [sic] of new clients”. Navigator B connected the high volume of requests to HCP and systemic factors, as well as the limits of peer navigation:

“Right now, our demand is so high and it’s really hard to keep up and assist everyone. This wouldn’t be an issue if it weren’t for the barriers in our systems. For example, I have clients needing mental health counsellors and family doctors in the city and rural areas. But there just isn’t anywhere I can send them right now!”

On the other hand, some unexpected work was perceived by the navigators as simply a product of trying to be the most effective at their positions that they could be. For example, the navigators found that establishing a social media presence greatly increased their reach and ability to connect with clients—and even HCPs. The navigators also became notaries public, in response to the unanticipated demand from clients for help with legal name and gender-marker changes. Navigator B reflected on the benefits of this: “I am able to assist folks with legal name changes if they haven’t been officially divorced from a spouse but cannot contact them for safety. At the beginning of the pilot, we didn’t know the answer on how to do this. No one did. But we figured it out!” The volume of requests for name and gender-marker changes was such that a collaboration with a local CBO took shape, and we see that the themes are intertwined, in this specific example, with teamwork and positive feelings.

#### 3.1.3. Teamwork

The theme of teamwork demonstrated the importance of the navigators’ collaborations with one another, while also reflecting the significant collaborations they established with HCPs and CBOs—one instance of the latter is outlined above. Mentorship from the trans peer navigators at Trans Care BC was something that both navigators appreciated, as Navigator B wrote:

“We’re really fortunate to have help and [support] from these nice folks. It’s admirable to see how successful their program is. They have so much organization in their services, and everything is so efficient and streamlined. Although we’re starting from ground zero in Saskatchewan, it’s nice to have something to aim towards as we build our program. The team at Trans Care BC is very inspiring!”

Teamwork like this also extended to being able to consult with HCPs on the research team.

The navigators also leaned on each other, which Navigator B connected to these previous mentorship opportunities: “Having another navigator to bounce ideas off is the reason I’m able to do this job. This job cannot be sustained without the connection of another navigator”. The navigators’ ability to connect with each other online was perceived as a key component to their success and sustainability throughout the pilot year and is reflected in the fact that the navigators conducted almost all their group education sessions together.

The navigators’ collaborations with CBOs could be helpful as well; for instance, Navigator A noted the myriad benefits for clients of the legal name change clinic they held in partnership with a CBO:

“We spoke with someone [who] is working with a team of law students to conduct a name change clinic and provide financial support for people [who] need financial support. This is going to be great because even for myself that the cost of doing a name change is steep for a piece of paper and a card. But this will be great because we can do this for people and there won’t be any mistakes because we will all know what is required to change markers.”

By working with this organization, the navigators were able to provide financial support for the applications of attendees who are TGD, and they were also able to help a higher volume of people at one time than they would otherwise have been able to do.

#### 3.1.4. Lived Experience

“Lived experience” reflects the almost universally positive impact of the navigators being TGD themselves. As Navigator A noted,

“Representation in working with the trans community is essential. It is always nerve wracking to seek support as a trans person because we often do not see trans people doing this work. We need people with lived experience to do these services because trust and translation are so important. If my clients didn’t trust me, I wouldn’t have achieved as much with each client. Translation is important because the health system is so complicated for community members; most people wouldn’t know about self-referrals if this service didn’t exist.”

Navigator B reported a specific example of the trust that the navigators were able to establish with clients:

“I found it quite interesting how quickly [a client] felt comfortable and safe with me. Within a few minutes into our call, they were opening up to me about their experiences with gender and revealing pieces of them that they reported not having told other people.”

Navigators having lived experience as people who are TGD helped clients communicate in a way they would not feel comfortable doing with someone who was cisgender, permitting the navigator to help the client more quickly and easily. Given that peer support and information were the most requested services, the impact of the navigators’ lived experience in their roles was crucial, since it allowed them to gain trust quickly, and gave them credibility in the eyes of clients.

The navigators’ lived experience also helped them in their group education sessions:

“The majority of our presentation covered case studies. Many of the cases related to personal experiences that [we] have had, which we know many of our community members have faced as well (Navigator B).”

They were able to lean on the professional literature and case studies, but also highlighted how these experiences were familiar to them personally and often representative of the TGD community’s experiences more generally.

#### 3.1.5. Healthcare Provider and Systemic Factors

Subthemes related to HCPs, or the healthcare system itself, were among the most interconnected of all—one of the “challenges” intersecting with this theme was the lack of physicians willing to provide transition-related care and the long waitlists to see those who were. Navigator A noted that “[t]he wait list[s] for most doctors, surgeons, psychologists are over a year and most cis health professionals and people in power don’t see this as an issue or something that needs to be address[ed] because they still see our [i.e., trans] healthcare as elective”. This quote exposes both an individual provider factor, with relatively few providers choosing to become proficient in transition-related care, and systemic factors, specifically that the healthcare system places low priority on trans healthcare needs as evidenced by not training providers and treating trans health as elective. As the year advanced the lack of physicians became even more noticeable, with the navigators having great difficulty finding primary care providers for their clients’ general (non-trans related) healthcare. Navigator B wrote: “it’s very frustrating though because as of right now, I have ZERO family doctors [for general primary care] to refer transgender people to”. The navigators became very frustrated by the combination of the limited number of knowledgeable, safe, and affirming HCPs and the very long wait times to see them, with the result that the navigators had no family physicians to refer clients to. Here, we see links to other subthemes in HCP and systemic factors, including HCPs’ education needs concerning TGD healthcare (and identities), the desire for all family physicians to be willing to provide hormone therapy, and a particular lack of mental healthcare providers who are safe and accessible for people who are TGD. All these subthemes in turn connect with other subthemes—particularly those identified as “challenges”.

Another HCP barrier for the navigators was not being recognised as professionals by others. Navigator B wrote:

“When I call clinics or reach out to professionals, I am not seen as important or credible. I am brushed off as if I’m just a nosy trans activist trying to do community advocacy. I wish my position was regarded more as healthcare role.”

This was experienced when contacting physicians’ offices, mental health clinics, CBOs, and other services. It may have been partly related to how busy everyone was dealing with the unusual demands created by COVID-19 or with the navigators being new and relatively unknown; however, the navigators believed that it had to do with not having an official home within the provincial healthcare system and, therefore, being seen as less legitimate.

The navigators recognised unequal funding for trans health services and a lack of services in the public health system as additional systemic barriers. For example, a double mastectomy is covered by provincial health insurance, but chest contouring and breast augmentation are not [14]. Hair removal and facial surgeries are not covered at all, so clients must pay out of pocket. Speech therapy to alter the pitch, tone, and timbre of the voice is covered if one sees a speech therapist in the public system; however, there are very few who practice in this area and they are only in the big cities:

“One thing that came up a few times this week was people needing voice therapy. We have two options in [city] and even if folks do voice therapy online, it is not covered and many of them can’t pay the fees.”

These systemic barriers affect trans men and women unequally, with the greater burden falling on trans women. The navigators were frustrated by not being able to help people more because of these structural barriers relating to insurance payments and the availability of HCPs.

As mentioned in the previous paragraph, services in Saskatchewan are concentrated in the two largest cities, leading to barriers for those who live in rural and remote areas. This exacerbated the overall lack of HCPs prepared to provide trans healthcare. Navigator B wrote: “I tweeted about the lack of trans healthcare and demand for providers in [rural] SK”. He received quite a few responses that was positive, but most were telling him about physicians in the nearest city who were already well known to the research team and not new HCPs wanting to learn about trans healthcare. This produced mixed emotions; it was encouraging to receive so many responses but discouraging to have so few interested in learning how to provide care to people who are so underserved. These are examples of some of the ways in which healthcare system and HCP factors generally served as barriers to the navigators’ work.

#### 3.1.6. Challenges

The theme of “challenges” then reflects the demanding nature of navigating on behalf of the TGD community, whose identities and needs are often not validated or understood, both in general society and within the medical community. As depicted in both Table 1 and Figure 1, the challenges were numerous; beyond the work of navigating, however, we can also highlight the particular challenge of how many HCPs perceived the navigators and their role. Working for a research project as part of a new pilot program and housed in CBOs as opposed to within the healthcare system, the navigators found that many HCPs questioned the legitimacy of their services and knowledge. For example, Navigator A noted that “Starting conversations with clinics is proving to be difficult. [Administrative staff] have never heard of [the CBO] before so they are not giving my messages to the managers”. This was a comment that, in some form or other, came up multiple times and led Navigator B to conclude that “[i]n the future of the Trans Navigation service, I hope it’s run through [the Saskatchewan Health Authority] rather than community organizations. I don’t think clinics and providers take [CBOs or people who work there] seriously or professionally”. Navigator B also remarked that “a certain client was not receptive to [Navigator A]’s services, likely because of pre-conceptions and feelings about the organization the navigation service is based out of”. We see, then, that both HCP and community opinions of the navigators was lessened by perceptions of CBOs.

Of course, this was far from the only challenge. Correcting misinformation, in both the TGD community and the healthcare system, was another challenge of some note. Navigator A noted early on during the year that for families seeking information concerning a child who is TGD there is a lot of “misinformation that is given to these parents by the media and even their family doctors”, and this could be quite harmful for these children. Writing later, Navigator B commented that “In the past, a lot of misinformation went around about how to access transition steps in online groups or social circles, but [now, c]ommunity members knew that they could just come to the navigators to get their answers”. Misinformation about transition and trans health was a significant concern for the navigators, but they also felt, after they had established themselves within the TGD community throughout Saskatchewan, that their work was able to overcome this to some extent, and they were looked to as authority figures.

Both navigators also noticed that clients with multiple marginalized identities (e.g., Indigenous, disabled) in addition to being TGD faced additional barriers. Navigator B wrote at length about an encounter with a neurodiverse client who sought a notarized signature for a name change form. The navigator had difficulty communicating because the client needed longer than usual to process information. He concluded the entry by writing: “Providers rush through their time with clients and don’t take into account the intersections with gender identity”. Elsewhere, both navigators wrote about how there were services for people with various individual identities, e.g., low income, neurodiverse, trans, Indigenous, but that these services were not able to accept people with multiple, intersecting marginalized identities, which led to clients facing a greater burden of discrimination or a lack of appropriate care.

## 4. Discussion

This study is novel because it examined the navigators’ experiences and not client outcomes or the efficacy of the program, which we assessed separately [9,10]. Overall, the themes we identified in the navigators’ journals tell us that their work was hard, yet rewarding. The sheer variety of tasks they took on was staggering, and they deftly switched between dealing with clients and HCPs. Though it was not the intent of this study, the navigators did reflect on their successes over time and clearly indicated that they thought they were helping to improve the lives of people who are TGD in Saskatchewan. In the end, the navigators perceived their success to be most related to being able to assist their clients, and they were proud of the things they were able to accomplish. We found limited literature analysing the experiences of navigators in other peer health navigation programs, which was why we undertook this study.

We could only find two other studies that discussed the experiences of healthcare navigators. Both were published after the TRANS Project completed its planning phase. Reid et al. interviewed ten pediatric patient navigators across Canada [3]. Seven were professional navigators, one was a peer navigator, and the other two were lay navigators. They found, similarly to our navigators, that lay models of navigation made it easier to develop a rapport with clients and that the navigators were not always respected by HCPs [3]. In the professional models of navigation they found, also similarly to our navigators, that system knowledge and understanding client needs are essential [3]. In Reid’s study, this came from professional training [3]; our navigators gained this knowledge from a combination of professional training and lived experience. Reid also found some commonalities between the lay and professional models [3]. First, that both fostered client-centred care and strove to meet people where they were, which were important goals for our navigators. Reid’s study found that being embedded in the healthcare system was an advantage [3], and our navigators identified that being outside the system was a barrier to their work. Finally, Reid found that the personality and experience of the navigators was important [3], which is very similar to our navigators who repeatedly commented on how much their lived experience helped them in their jobs. The most significant differences between Reid’s study and ours were that it employed interviews, rather than journals, which may have introduced greater recall bias; it involved a greater number of navigators, from a wider variety of contexts, which may increase the applicability of the findings to other settings, and the navigators were not included in the research team, which may limit the insight of the research, as key intuitions may have been missed by not including navigators as researchers.

Gauthier et al. conducted a pilot study with lay navigators in primary care in Sudbury and Ottawa, Ontario, Canada [6]. They had two navigators reflectively journal about critical learning incidents; together they recorded a total of 66 journal entries [6]. The research explored the navigators’ learning experiences and their implications for education and health promotion. They found five themes; four of which were very similar to ours and one which differed. The similar themes were as follows: Gaining and Building Trust, Experiencing Hope and Optimism, Feeling Helpless, and Celebrating Gains and Successes [6]. They found that gaining and building trust was an ongoing process that lasted for the entire duration of the intervention with each client, which contrasted with our navigators who found that they built trust quickly. Similarly to our navigators, Gauthier’s navigators experienced hope and optimism when clients demonstrated motivation to help themselves and followed through with recommendations. Both Gauthier’s and our navigators felt helpless in very similar circumstances, when up against systemic obstacles. There were some additional differences, Gauthier’s navigators came up against barriers posed by the study design and frustration when clients lacked motivation and follow through, while our navigators were limited by working outside the healthcare system. Both sets of navigators took huge pleasure in celebrating even small improvements or achievements for their clients. The theme that was most different form our results was “Gaining and Building Trust” [6]. Our navigators wrote about how easily trust developed between them and their clients, contrasting with the navigators in the Gauthier study. A possible reason for this difference is that our navigators shared more of their lived experience with their clients which facilitated a rapport and trust and they were part of the research team which gave them more control over their work environment, whereas the navigators in Gauthier’s study did not share the lived experience of their clients and were subject to limitations imposed by the study design over which they had no control because they were not researchers. The research questions were also quite different between the two studies, which is likely to have been part of the different emphases in the findings. Despite their differences, the similarities between our study, Reid’s, and Gauthier’s are remarkable. This suggests that we have found some characteristics of healthcare navigation that are transferable across settings, at least within Canada, such as the importance of a rapport, empathy, and lived experience, that navigators who work outside the healthcare system are not always respected by HCPs, that systemic barriers impede navigators’ work, and that clients’ successes make the job rewarding.

The navigators consistently noted that performing this work as people who are TGD themselves, who functioned as advocates for members of and on behalf of their own community, was part of their success and helped them to establish rapports with clients, as well as providing them with a greater understanding of why people who are TGD might seek care and the type of care that they hoped to receive. This was anticipated as the rationale for employing peer navigation—to lean on the relatability and expertise of people with lived expertise who represent a community. It provided meaning to the navigators and made the work particularly rewarding to them, and they themselves identified this as a factor that they believed contributed to their successes.

Barriers to their work were perceived as both frustrating and, in some cases, unnecessary. A lack of affirming HCPs willing to provide trans-specific care led to difficulty in helping clients access care or caused substantial delays. The navigators consistently highlighted that resistance from HCPs to providing trans-specific care hindered their work and were based on HCPs’ personal choices as opposed to strictly medical reasons. Christopherson et al. studied Saskatchewan HCPs’ comfort and willingness with providing trans-specific care and found that 66.5% (n = 125) of the 188 Saskatchewan HCPs surveyed indicated that they had no formal training with transition-related healthcare [2]. Only 30.3% (n = 57) of respondents indicated that they were comfortable providing transition-related medical care [2]. The researchers also noted that 9.6% of respondents (n = 18) indicated that they had “ethical, religious, or moral reservations about providing transition-related healthcare” [2] (p. 471). Since Christopherson et al. also found that most participants (76%, n = 142) were interested in education on trans-specific healthcare needs [2], training must be prioritized—given that the lack of primary HCPs was so frequently discussed in the journals.

Another challenge for the navigators that created a barrier to effectively conducting their work was the perceived legitimacy of their positions, which operated outside of Saskatchewan’s formal healthcare system. This was a by-product of their positions being piloted by a university research team, as opposed to by the healthcare system. Their journals reflected a very real perception that working from within the healthcare system would have increased the perceived legitimacy of their work in the eyes of HCPs and that being based in CBOs reduced their credibility. As healthcare and health needs were the primary focus of their work with and on behalf of people who are TGD, the navigators identified a desire for their work to be acknowledged as healthcare work and that future navigation work in Saskatchewan on behalf of the TGD community would be best accomplished by fully integrating these positions within the healthcare system itself.

We found only one other study that addressed the perceived legitimacy of navigators as part of the healthcare team or how their “homebase” affected their credibility among other HCPs [3]. Another pilot study noted the limits of their navigators’ work simply as a by-product of being a pilot [15]. We also note that, overall, it seems to be unusual for navigators to be housed outside formal healthcare systems [4,6,16,17,18]. This suggests that being based out of a CBO was a barrier. Here, we identify that more research of the experiences of navigators themselves is needed, beyond merely evaluating the efficacy of their positions. In British Columbia in particular, peer navigators for people who are TGD *are* housed in the provincial health authority [4], so there is another Canadian model supporting what our navigators perceived as the most effective way to conduct their work, although a published formal evaluation of this provincial program’s efficacy has not been undertaken.

### 4.1. Strengths and Limitations

The journaling method produced insights that could not have been obtained through retrospective interviews. We were able to analyze how the navigators felt about issues and topics as they arose. We were able to see how the navigators felt about their own work, including what was clearly a high workload and a high-stress, if high-reward, working environment. We were also able to identify improvements and recommendations from the navigators to improve their quality of work or their ability to do their jobs. Analyzing the journals together at the end of the project gave the navigators insights, such as what they had learned over time and how they had incorporated past experiences into present practice over the course of the pilot year. Our large analysis team, which included the navigators, was strong because it allowed us to include people who are TGD throughout the process without unduly adding to minority stress; it allowed us to assign researchers who did not live in the same city as the navigator to the smaller analysis teams to protect anonymity, and it gave us the benefit of the navigators’ deep understanding of their roles when analyzing the journals.

We acknowledge the limitations present in the methods as well: having the navigators participate in the analysis may have caused other researchers on the team to self-censor during discussions or may have favoured the navigators’ own interpretations of their experiences. However, we were a well-established research team, since 2018, with norms that promoted open, respectful discussions, encouraged expressions of disagreement, and welcomed questions. Including only the two journals as data did not allow for a triangulation analysis with outside data which may have limited the validity of our findings. We also only performed a limited analysis (comparing and contrasting) of the differences between themes between the two navigators’ journals. However, given that the purpose of this research was not to find truth, but rather to make meaning [19] (p. 238), having a large analysis team that worked by consensus worked to ensure that there was a shared understanding of the experience of being navigators that was not unduly influenced by any individual’s bias. Including only two journals, from only one navigator program in one small Canadian province for one pilot year, does suggest a certain homogeneity in experiences between the two navigators given how closely they worked together and a limitation in the variability of the experiences. Thus, there is no way to know if saturation was reached. It also limits the transferability of these findings. It is our intention to use these results as a foundation to study the experiences of trans healthcare navigators across Canada to better understand the differences and similarities among services and how they affect navigators’ work.

### 4.2. Implications

There are several implications from this work. The first is a better understanding of the complexity of our healthcare navigators for TGD people’s work in terms of the number of tasks that they performed, the diversity of these tasks, and the emotional demands of the work. Better understanding the actual requirements of the work allowed us to provide better support to the navigators as the pilot was running, e.g., emotional support, continuing education, and on-the-job training and mentorship. It may assist others who are establishing navigator programs to anticipate their navigators’ needs and to have supports, benefits, including adequate coverage for mental healthcare and time off, procedures for collaborations across sites with counterparts, and education in place earlier than we did, which is likely to reduce the stress on the peer navigators.

The second major implication is that understanding the network of the navigators’ responsibilities and how they interact with HCPs, CBOs, and other service providers in the community allows for a focus on relationship building. This may prevent the difficulties we experienced with the navigators not being taken seriously by some HCPs and the turf conflicts they had early on with some 2SLGBTQ+ CBOs. Stronger relationships would both reduce the stress on the navigators and improve services for clients, as there would be less duplication of services, a better flow for clients, and HCPs might be more likely to reach out to the navigators for education, which would increase the number of providers available to provide care to people who are TGD.

## 5. Conclusions

This research is novel as it is the first to look at the experience of being a trans peer navigator from the perspective of the navigator. We found that it is simultaneously a very rewarding and extremely challenging job. The rewards came from being able to meaningfully help people in their community. The challenges came from not being respected by other HCPs and from systemic barriers, like a lack of physicians willing to provide trans-related care, that prevented them from providing the assistance their clients needed. The navigators were able to adapt their services to bridge some systemic barriers and to provide insightful recommendations for remediating others. We recommend that policymakers build policies and procedures around navigators’ wellbeing, the diversity of the work, teamwork, and the longevity of funding if developing similar programs. We also recommend that similar programs be housed within the healthcare system.

## Figures and Tables

**Table 1:** The themes that emerged from the navigators’ journals. Tasks are key examples of the types of work in each theme, with examples provided for clarification as needed.

Themes	Tasks
1. Expected work— work that was anticipated by the research team when planning the intervention	Assisting with access to hormone therapy, including blockers
	Addressing misinformation
	Education—provided by navigators, and navigators’ education needs
	Creating resources
	Connecting MDs to mentors
	Facilitating systemic change and increased capacity
	Peer support
	Networking with CBOs
	Managing clients’ expectations
	Ensuring clients’ safety in interactions with navigators
	Suicide prevention
2. Unexpected work—work that emerged as important during the intervention	High volume of email/contacts
	Effects of COVID-19 on work
	Education—the volume of education provided and the variety of audiences were unexpected.
	Education—received
	Legal clinics—name and gender marker
	Difficulty finding affirming HCPs
	Finding funding for clients/free clinics for services not covered by provincial health insurance: e.g., laser hair removal and many mental healthcare services
	Receiving donations of gender affirming products: e.g., binders and gaffs
	Social media/communication: it was not anticipated that it would be the most effective way to communicate with HCPs
3. Teamwork—the high degree to which the navigators’ work depended upon working collaboratively with each other and in partnership with local, regional, and other trans-focused CBOs	Communication between the navigators
	Expectations for attending training/meetings at hosting CBO
	Mentorship from other navigators from outside of SK
	Creating legal change clinic
	CBOs have very limited capacity to financially support people
	Networking in health system
	CBOs provide safe mental health services
	Hormone injections provided by sexual health CBO
	Defining roles with other CBOs to avoid duplicating services
	Publicity and referrals from CBOs
	Lack of support when CBO is reorganizing
	Education sessions with non-2SLGBTQ+ CBOs
4. Lived experience—the importance of being trans themselves for working effectively as navigators	Role change from trans person to navigator
	Questioning how own experience affects expectations for clients
	Pre-existing understanding of community needs and available services
	Lived experience is preparation for navigator job
	Positive reactions from clients and parents
	Trusted because of lived experience
	High expectations of self and from others
	Small province and trans community; therefore, must share support spaces with clients
	Rural and older clients appreciate connection to other trans people
5. Challenges—factors that made the jobs more complicated and demanded more emotional energy	Lack of funding: e.g., mental healthcare, aesthetic services
	Helping people access hormone therapy, including hormone blockers
	Lack of HCPs, both those able to provide trans-specific care and generally
	COVID-19 restrictions
	Addressing misinformation
	Communicating with HCPs
	Education sessions that go poorly: e.g., transphobic reactions, HCPs attached to outdated standards of trans care
	Lack of trans-affirming mental healthcare providers
	High expectations from clients
	Lack of support from hosting CBOs
	Lack of support and resources for TGD youth
	Clients’ prior negative experiences with 2SLBGTQ+ CBOs made them less willing to work with navigators
	Clients not knowing/not being able to articulate what they needed/wanted and refusing/being dissatisfied with what the navigators were able to offer
	Intersectionality of multiple marginalized identities
	Clients reporting that MDs were dismissive and transphobic
	Backlash from being a public-facing trans person
6. Healthcare provider (HCP) and systemic factors —Environmental factors that shaped the navigators’ work.	Unequal funding and lack of services in the public healthcare system
	Navigators facilitated HCPs work: e.g., by explaining things to clients, by explaining referral processes to both clients and HCPs
	Variability in providers’ ability to support youth on their gender journey
	Lack of family MDs to provide hormone therapy and safe care
	Lack of affirming mental healthcare providers
	Lack of perceived legitimacy of navigators by HCPs
	Waitlists to see affirming HCPs
	Many HCPs need education on TGD healthcare
	Rushed healthcare appointments
	Navigator job insecurity
	Unequal distribution of services (HCPs and CBOs) across the province
	Need for increased community supports, e.g., services for youth, funding for uninsured services, gender affirming products, services for people with multiple stigmatized identities

Key: CBOs: community-based organizations; HCPs: healthcare providers; MD: medical doctor; SK: Saskatchewan; TGD: trans and gender diverse; 2SLGBTQ+: Two Spirit, lesbian, gay, bisexual, transgender, queer, and other identities under the queer umbrella.

## Data Availability

Due to the sensitive and personal nature of the data related to this project, they are not publicly available.

## Abbreviations

The following abbreviations are used in this manuscript:

TGD	Transgender or gender-diverse
HPC	Healthcare provider
CBO	Community-based organisation
2SLGBTQ+	Two Spirit, lesbian, gay, bisexual, transgender, queer, plus other identities
